# 5-{2-(4-Chloro­phen­yl)-1-[2-(4-chloro­phen­yl)-1-(3,4,5-trimeth­oxy­phen­yl)eth­oxy]eth­yl}-1,2,3-trimeth­oxy­benzene

**DOI:** 10.1107/S1600536812008823

**Published:** 2012-03-10

**Authors:** Ying Fu, Mu Yuan, Xuemei Hu, Yanshou Yang, Hongxia Hou

**Affiliations:** a College of Chemistry and Chemical Engineering, Northwest Normal University, Lanzhou, Gansu Province 730070, People’s Republic of China

## Abstract

The title compound, C_34_H_36_Cl_2_O_7_, is a by-product from the reaction of 4-chloro­benzyl­zinc chloride with 3,4,5-trimeth­oxy­benzaldehyde. In each of the two 1,2-diphenyl­ethyl moieties, the two benzene rings are arranged in a *trans* conformation and make C_ar_—C—C—C_ar_ torsion angles of 163.64 (19) and 174.43 (18)°. The crystal structure is stabilized by van der Waals inter­actions only.

## Related literature

For the synthesis and reaction of organozinc reagents, see: Rappoport & Marek (2007 ▶); Knochel & Jones (1999 ▶); Erdik (1996 ▶); Knochel (2005 ▶). For the synthesis of diphenyl­ethyl ether, see: Lenselink & Johan van Manen (2001 ▶). For the structure of anisole, see: Seip & Seip (1973 ▶).
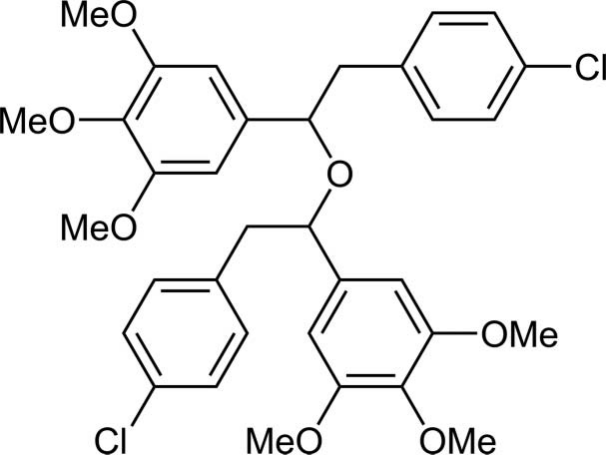


## Experimental

### 

#### Crystal data

C_34_H_36_Cl_2_O_7_
*M* *_r_* = 627.53
Monoclinic, 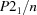

*a* = 13.3326 (8) Å
*b* = 13.5487 (8) Å
*c* = 18.1703 (11) Å
β = 100.036 (3)°
*V* = 3232.0 (3) Å^3^
*Z* = 4
Mo *K*α radiation
μ = 0.25 mm^−1^
*T* = 296 K
0.38 × 0.35 × 0.34 mm

#### Data collection

Bruker APEXII CCD diffractometer
Absorption correction: multi-scan (*SADABS*; Bruker, 2004 ▶) *T*_min_ = 0.912, *T*_max_ = 0.921
16506 measured reflections
5707 independent reflections
3682 reflections with *I* > 2σ(*I*)
*R*_int_ = 0.036

#### Refinement

*R*[*F* ^2^ > 2σ(*F* ^2^)] = 0.043
*wR*(*F* ^2^) = 0.116
*S* = 1.05
5707 reflections
395 parameters
H-atom parameters constrained
Δρ_max_ = 0.20 e Å^−3^
Δρ_min_ = −0.27 e Å^−3^

### 

Data collection: *APEX2* (Bruker, 2004 ▶); cell refinement: *SAINT-Plus* (Bruker, 2004 ▶); data reduction: *SAINT-Plus*; program(s) used to solve structure: *SHELXS97* (Sheldrick, 2008 ▶); program(s) used to refine structure: *SHELXL97* (Sheldrick, 2008 ▶); molecular graphics: *SHELXTL* (Sheldrick, 2008 ▶); software used to prepare material for publication: *SHELXTL*.

## Supplementary Material

Crystal structure: contains datablock(s) I, global. DOI: 10.1107/S1600536812008823/bx2396sup1.cif

Structure factors: contains datablock(s) I. DOI: 10.1107/S1600536812008823/bx2396Isup2.hkl

Supplementary material file. DOI: 10.1107/S1600536812008823/bx2396Isup3.mol

Supplementary material file. DOI: 10.1107/S1600536812008823/bx2396Isup4.cml

Additional supplementary materials:  crystallographic information; 3D view; checkCIF report

## Figures and Tables

**Fig. 1.**
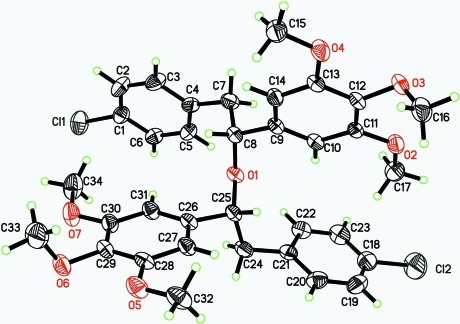
A view of the molecule with the atom numbering scheme. Displacement ellipsoids are drawn at the 30% probability level.

## supplementary crystallographic information

### Comment

The reaction of organometallic reagents with aldehydes gave many by-products owing to the basicity of organometallic reagents and acidity of their metallic salts. The title compound, Fig. 1, C_34_H_36_Cl_2_O_7_, was isolated as a by-product of 4-chlorobenzylzinc chloride with 3,4,5-trimethoxybenzaldehyde in less than five percent. All four aromatic rings of (I) are planar, with a maximum deviation of 0.022 (2) Å for atom C13 and C27 from the least-squares plane defined by atoms C9–C14 and C26—C31 respectivey. The mean C_aryl_–O bond [1.370 Å] is slightly larger than a normal C–O single-bond distance for anosole (1.357 Å, Seip & Seip, 1973), implying the steric hindrance of the *ortho* neighbouring three methoxy groups in one phenyl ring slacked down the conjugations between the methoxy groups and the aromatic ring. In each of the two 1,2-diphenyl ethane moiety, the two phenyl rings are arranged in a *trans* conformation as the dihedral angle of C4—C7—C8—C9 and C21—C24—C25—C26 was found to be 174.43 (18)° and 163.64 (19) ° respectively. In the crystal structure, there are no classic hydrogen bonds and no significant intermolecular π–π interactions between the molecules.

### Experimental

Under the nitrogen atmosphere, 3,4,5-trimethoxybenzaldehyde (0.37 g, 1.89 mmol) and trimethylsilyl chloride(0.48 ml, 3.78 mmol) in THF (10 ml) was added a solution of *p*-Chlorobenzyl zinc chloride reagents (1.0 *M*, 2.5 ml) cooled with ice-water bath. The reaction was stirred for 12 h at room temperature, then quenched with 10 ml of 1.0 *M* HCl. After the usual work up, the title compound was isolated in 5% yield as white solid, mp: 156–158 *^o^*C. IR (KBr), *v* (cm^-1^): 2926, 1593, 1500, 1460, 1421, 1350, 1325, 1233, 1180, 1128, 1079, 1010; ^1^H NMR (400 MHz, CDCl_3_) δ (p.p.m.): 2.71 (dd, *J* = 13.6, 3.5 Hz, 2H, CH_2_), 2.91 (dd, *J* = 13.6, 3.5 Hz, 2H, CH_2_), 3.62 (s, 12H, OCH_3_), 3.83 (s, 6H, OCH_3_), 4.15 (dd, *J* = 9.1, 3.6 Hz, 2H, CH), 6.09 (s, 4H, ArH), 7.12 (d, *J* = 8.4 Hz, 4H, ArH), 7.25 (d, *J* = 8.8 Hz, 4H, ArH); ^13^C NMR (100 MHz, CDCl_3_): 153.16, 137.29, 137.08, 137.05, 132.07, 131.36, 127.99, 103.25, 79.07, 60.81, 55.81, 44.57; ESI-MS m/*z* (*M* + NH_4_^+^): 644.2243.

### Refinement

All H atoms were geometrically positioned and refined using a riding model with C—H = 0.93 Å, *U*_iso_(H) = 1.2 *U*_eq_(C) for aromatic atoms, C—H = 0.98 Å, *U*_iso_(H) = 1.2 *U*_eq_(C) for CH atoms, C—H = 0.97 Å, *U*_iso_(H) = 1.2 *U*_eq_(C) for CH_2_ atoms and C—H = 0.96 Å, *U*_iso_(H) = 1.5 *U*_eq_(C) for CH_3_ atoms.

### Crystal data

**Table:**

C_34_H_36_Cl_2_O_7_	*F*(000) = 1320
*M**_r_* = 627.53	*D*_x_ = 1.290 Mg m^−^^3^
Monoclinic, *P*2_1_/*n*	Melting point: 429 K
Hall symbol: -P 2yn	Mo *K*α radiation, λ = 0.71073 Å
*a* = 13.3326 (8) Å	Cell parameters from 3179 reflections
*b* = 13.5487 (8) Å	θ = 2.3–21.4°
*c* = 18.1703 (11) Å	µ = 0.25 mm^−^^1^
β = 100.036 (3)°	*T* = 296 K
*V* = 3232.0 (3) Å^3^	Block, colourless
*Z* = 4	0.38 × 0.35 × 0.34 mm

### Data collection

**Table:**

Bruker APEXII CCD diffractometer	5707 independent reflections
Radiation source: fine-focus sealed tube	3682 reflections with *I* > 2σ(*I*)
Graphite monochromator	*R*_int_ = 0.036
φ and ω scans	θ_max_ = 25.1°, θ_min_ = 1.8°
Absorption correction: multi-scan (*SADABS*; Bruker, 2004)	*h* = −15→15
*T*_min_ = 0.912, *T*_max_ = 0.921	*k* = −16→16
16506 measured reflections	*l* = −19→21

### Refinement

**Table:**

Refinement on *F*^2^	Secondary atom site location: difference Fourier map
Least-squares matrix: full	Hydrogen site location: inferred from neighbouring sites
*R*[*F*^2^ > 2σ(*F*^2^)] = 0.043	H-atom parameters constrained
*wR*(*F*^2^) = 0.116	*w* = 1/[σ^2^(*F*_o_^2^) + (0.0488*P*)^2^ + 0.3898*P*] where *P* = (*F*_o_^2^ + 2*F*_c_^2^)/3
*S* = 1.05	(Δ/σ)_max_ < 0.001
5707 reflections	Δρ_max_ = 0.20 e Å^−^^3^
395 parameters	Δρ_min_ = −0.27 e Å^−^^3^
0 restraints	Extinction correction: *SHELXL97* (Sheldrick, 2008), Fc^*^=kFc[1+0.001xFc^2^λ^3^/sin(2θ)]^-1/4^
Primary atom site location: structure-invariant direct methods	Extinction coefficient: 0.0023 (5)

### Special details

**Table:**

Geometry. All e.s.d.'s (except the e.s.d. in the dihedral angle between two l.s. planes) are estimated using the full covariance matrix. The cell e.s.d.'s are taken into account individually in the estimation of e.s.d.'s in distances, angles and torsion angles; correlations between e.s.d.'s in cell parameters are only used when they are defined by crystal symmetry. An approximate (isotropic) treatment of cell e.s.d.'s is used for estimating e.s.d.'s involving l.s. planes.
Refinement. Refinement of *F*^2^ against ALL reflections. The weighted *R*-factor *wR* and goodness of fit *S* are based on *F*^2^, conventional *R*-factors *R* are based on *F*, with *F* set to zero for negative *F*^2^. The threshold expression of *F*^2^ > σ(*F*^2^) is used only for calculating *R*-factors(gt) *etc*. and is not relevant to the choice of reflections for refinement. *R*-factors based on *F*^2^ are statistically about twice as large as those based on *F*, and *R*- factors based on ALL data will be even larger.

### Fractional atomic coordinates and isotropic or equivalent isotropic displacement parameters (Å^2^)

**Table:**

	*x*	*y*	*z*	*U*_iso_*/*U*_eq_	
C1	0.58651 (18)	0.79978 (18)	0.07380 (12)	0.0542 (6)	
C2	0.64191 (19)	0.7228 (2)	0.10839 (14)	0.0687 (7)	
H2	0.7093	0.7136	0.1028	0.082*	
C3	0.59641 (19)	0.65910 (19)	0.15159 (14)	0.0643 (7)	
H3	0.6341	0.6071	0.1757	0.077*	
C4	0.49592 (17)	0.67065 (16)	0.15990 (12)	0.0491 (6)	
C5	0.44157 (17)	0.74770 (17)	0.12206 (12)	0.0534 (6)	
H5	0.3734	0.7561	0.1257	0.064*	
C6	0.48656 (18)	0.81213 (18)	0.07916 (12)	0.0549 (6)	
H6	0.4491	0.8636	0.0540	0.066*	
C7	0.44753 (19)	0.60401 (17)	0.21008 (12)	0.0554 (6)	
H7A	0.4820	0.5407	0.2136	0.067*	
H7B	0.3770	0.5929	0.1874	0.067*	
C8	0.45094 (16)	0.64477 (15)	0.28885 (11)	0.0441 (5)	
H8	0.5216	0.6603	0.3108	0.053*	
C9	0.41014 (16)	0.56853 (15)	0.33698 (11)	0.0422 (5)	
C10	0.30679 (16)	0.56178 (15)	0.33682 (11)	0.0449 (5)	
H10	0.2624	0.6078	0.3108	0.054*	
C11	0.26946 (15)	0.48582 (15)	0.37567 (12)	0.0430 (5)	
C12	0.33501 (16)	0.41591 (14)	0.41364 (12)	0.0438 (5)	
C13	0.43857 (16)	0.42317 (15)	0.41310 (12)	0.0439 (5)	
C14	0.47611 (16)	0.50010 (15)	0.37584 (12)	0.0458 (5)	
H14	0.5459	0.5059	0.3769	0.055*	
C15	0.60334 (17)	0.35426 (18)	0.45263 (14)	0.0632 (7)	
H15A	0.6178	0.3543	0.4027	0.095*	
H15B	0.6354	0.2982	0.4793	0.095*	
H15C	0.6292	0.4138	0.4777	0.095*	
C16	0.2916 (2)	0.34587 (19)	0.52286 (14)	0.0718 (8)	
H16A	0.3593	0.3527	0.5508	0.108*	
H16B	0.2601	0.2885	0.5398	0.108*	
H16C	0.2524	0.4033	0.5301	0.108*	
C17	0.09810 (17)	0.54094 (18)	0.34035 (14)	0.0642 (7)	
H17A	0.1170	0.6067	0.3569	0.096*	
H17B	0.0312	0.5264	0.3502	0.096*	
H17C	0.0982	0.5359	0.2877	0.096*	
C18	0.00522 (19)	0.77869 (19)	0.31385 (18)	0.0693 (7)	
C19	0.0580 (2)	0.8121 (2)	0.38014 (16)	0.0723 (8)	
H19	0.0268	0.8159	0.4220	0.087*	
C20	0.15810 (19)	0.84040 (19)	0.38457 (14)	0.0638 (7)	
H20	0.1939	0.8635	0.4298	0.077*	
C21	0.20640 (17)	0.83515 (15)	0.32347 (13)	0.0499 (6)	
C22	0.1507 (2)	0.80016 (17)	0.25690 (13)	0.0594 (6)	
H22	0.1817	0.7953	0.2150	0.071*	
C23	0.0503 (2)	0.77252 (19)	0.25182 (15)	0.0691 (7)	
H23	0.0135	0.7499	0.2067	0.083*	
C24	0.31432 (17)	0.87007 (16)	0.32892 (14)	0.0574 (6)	
H24A	0.3268	0.9204	0.3674	0.069*	
H24B	0.3209	0.9012	0.2819	0.069*	
C25	0.39760 (16)	0.79170 (15)	0.34644 (12)	0.0450 (5)	
H25	0.3824	0.7495	0.3869	0.054*	
C26	0.49995 (16)	0.84196 (14)	0.37206 (12)	0.0436 (5)	
C27	0.52939 (17)	0.85910 (15)	0.44770 (12)	0.0489 (6)	
H27	0.4907	0.8339	0.4813	0.059*	
C28	0.61632 (17)	0.91369 (15)	0.47393 (12)	0.0474 (6)	
C29	0.67365 (16)	0.95157 (15)	0.42418 (13)	0.0487 (6)	
C30	0.64530 (17)	0.93266 (17)	0.34829 (13)	0.0512 (6)	
C31	0.55848 (17)	0.87795 (16)	0.32219 (12)	0.0503 (6)	
H31	0.5397	0.8655	0.2713	0.060*	
C32	0.6009 (2)	0.88780 (18)	0.60143 (13)	0.0688 (7)	
H32A	0.5333	0.9142	0.5967	0.103*	
H32B	0.6376	0.9010	0.6507	0.103*	
H32C	0.5973	0.8178	0.5932	0.103*	
C33	0.8490 (2)	0.9565 (2)	0.47062 (18)	0.0942 (10)	
H33A	0.8401	0.9082	0.5076	0.141*	
H33B	0.9027	1.0010	0.4909	0.141*	
H33C	0.8663	0.9239	0.4276	0.141*	
C34	0.6939 (2)	0.9476 (2)	0.22823 (14)	0.0849 (9)	
H34A	0.6928	0.8771	0.2227	0.127*	
H34B	0.7482	0.9746	0.2061	0.127*	
H34C	0.6301	0.9744	0.2037	0.127*	
Cl1	0.64340 (6)	0.88568 (6)	0.02261 (4)	0.0871 (3)	
Cl2	−0.12145 (6)	0.74137 (8)	0.30797 (7)	0.1272 (4)	
O1	0.39285 (10)	0.73366 (10)	0.28040 (7)	0.0437 (4)	
O2	0.16884 (11)	0.47272 (11)	0.37913 (9)	0.0572 (4)	
O3	0.29618 (11)	0.33562 (10)	0.44572 (9)	0.0547 (4)	
O4	0.49673 (11)	0.34905 (11)	0.44998 (9)	0.0583 (4)	
O5	0.65201 (12)	0.93281 (12)	0.54773 (8)	0.0618 (4)	
O6	0.75737 (12)	1.01001 (12)	0.44949 (9)	0.0613 (4)	
O7	0.70919 (13)	0.97192 (13)	0.30468 (9)	0.0732 (5)	

### Atomic displacement parameters (Å^2^)

**Table:**

	*U*^11^	*U*^22^	*U*^33^	*U*^12^	*U*^13^	*U*^23^
C1	0.0542 (15)	0.0664 (16)	0.0440 (14)	0.0002 (12)	0.0146 (11)	0.0012 (12)
C2	0.0510 (15)	0.090 (2)	0.0694 (18)	0.0160 (14)	0.0234 (13)	0.0124 (16)
C3	0.0636 (17)	0.0661 (17)	0.0662 (17)	0.0237 (13)	0.0196 (13)	0.0127 (14)
C4	0.0578 (15)	0.0469 (13)	0.0434 (13)	0.0038 (11)	0.0113 (11)	−0.0050 (11)
C5	0.0444 (13)	0.0629 (16)	0.0539 (15)	0.0078 (12)	0.0110 (11)	0.0012 (12)
C6	0.0586 (15)	0.0573 (15)	0.0495 (14)	0.0104 (12)	0.0113 (12)	0.0056 (12)
C7	0.0700 (16)	0.0436 (13)	0.0540 (14)	−0.0026 (11)	0.0147 (12)	−0.0045 (11)
C8	0.0464 (12)	0.0372 (12)	0.0476 (13)	−0.0048 (10)	0.0050 (10)	0.0000 (10)
C9	0.0484 (13)	0.0340 (12)	0.0430 (12)	−0.0074 (10)	0.0043 (10)	−0.0030 (10)
C10	0.0500 (14)	0.0339 (12)	0.0487 (13)	−0.0029 (10)	0.0026 (10)	−0.0014 (10)
C11	0.0404 (12)	0.0355 (12)	0.0530 (14)	−0.0065 (10)	0.0074 (10)	−0.0074 (10)
C12	0.0509 (13)	0.0296 (11)	0.0502 (13)	−0.0090 (10)	0.0067 (11)	−0.0001 (10)
C13	0.0482 (13)	0.0323 (11)	0.0490 (13)	−0.0030 (10)	0.0021 (10)	−0.0005 (10)
C14	0.0429 (12)	0.0406 (12)	0.0529 (14)	−0.0065 (10)	0.0053 (10)	−0.0001 (11)
C15	0.0488 (14)	0.0591 (16)	0.0793 (18)	0.0039 (12)	0.0040 (13)	0.0126 (14)
C16	0.0837 (19)	0.0633 (17)	0.0715 (19)	−0.0082 (14)	0.0221 (15)	0.0152 (14)
C17	0.0507 (14)	0.0664 (17)	0.0731 (18)	0.0049 (13)	0.0037 (13)	0.0028 (14)
C18	0.0493 (15)	0.0597 (17)	0.096 (2)	0.0093 (12)	0.0044 (16)	0.0031 (16)
C19	0.0670 (18)	0.0773 (19)	0.077 (2)	0.0128 (15)	0.0239 (15)	−0.0019 (16)
C20	0.0624 (17)	0.0695 (17)	0.0575 (16)	0.0079 (13)	0.0050 (13)	−0.0139 (13)
C21	0.0559 (14)	0.0367 (12)	0.0557 (15)	0.0049 (11)	0.0060 (12)	−0.0006 (11)
C22	0.0726 (18)	0.0544 (15)	0.0496 (15)	0.0030 (13)	0.0061 (13)	0.0017 (12)
C23	0.0664 (18)	0.0681 (18)	0.0639 (18)	0.0009 (14)	−0.0134 (14)	−0.0007 (14)
C24	0.0633 (16)	0.0393 (13)	0.0685 (16)	−0.0038 (11)	0.0080 (12)	−0.0051 (12)
C25	0.0564 (14)	0.0355 (12)	0.0427 (13)	−0.0089 (10)	0.0080 (10)	−0.0005 (10)
C26	0.0531 (13)	0.0327 (11)	0.0433 (13)	−0.0051 (10)	0.0034 (10)	−0.0005 (10)
C27	0.0622 (15)	0.0404 (13)	0.0442 (14)	−0.0090 (11)	0.0098 (11)	0.0012 (10)
C28	0.0628 (15)	0.0357 (12)	0.0407 (13)	−0.0040 (11)	0.0009 (11)	−0.0045 (10)
C29	0.0509 (14)	0.0396 (13)	0.0535 (15)	−0.0105 (10)	0.0030 (11)	−0.0046 (11)
C30	0.0548 (14)	0.0477 (14)	0.0522 (15)	−0.0105 (11)	0.0121 (11)	0.0010 (11)
C31	0.0604 (15)	0.0488 (14)	0.0405 (13)	−0.0106 (12)	0.0054 (11)	−0.0014 (11)
C32	0.107 (2)	0.0524 (15)	0.0450 (15)	−0.0135 (14)	0.0090 (14)	−0.0014 (12)
C33	0.0593 (18)	0.092 (2)	0.123 (3)	−0.0005 (16)	−0.0083 (17)	−0.014 (2)
C34	0.079 (2)	0.125 (3)	0.0535 (18)	−0.0234 (18)	0.0178 (14)	0.0025 (17)
Cl1	0.0836 (5)	0.1034 (6)	0.0790 (5)	−0.0143 (4)	0.0275 (4)	0.0218 (4)
Cl2	0.0560 (5)	0.1255 (8)	0.1971 (11)	−0.0035 (5)	0.0139 (6)	−0.0086 (7)
O1	0.0557 (9)	0.0342 (8)	0.0393 (8)	−0.0030 (7)	0.0033 (7)	0.0004 (6)
O2	0.0449 (9)	0.0475 (9)	0.0795 (12)	−0.0018 (7)	0.0112 (8)	0.0082 (8)
O3	0.0594 (10)	0.0386 (9)	0.0657 (11)	−0.0121 (7)	0.0099 (8)	0.0064 (8)
O4	0.0500 (9)	0.0445 (9)	0.0783 (11)	0.0003 (7)	0.0051 (8)	0.0197 (8)
O5	0.0810 (12)	0.0581 (10)	0.0434 (10)	−0.0171 (9)	0.0028 (8)	−0.0069 (8)
O6	0.0544 (10)	0.0563 (10)	0.0697 (11)	−0.0166 (8)	0.0012 (8)	−0.0085 (9)
O7	0.0747 (12)	0.0884 (13)	0.0591 (11)	−0.0345 (10)	0.0185 (9)	−0.0040 (10)

### Geometric parameters (Å, º)

**Table:**

C1—C6	1.363 (3)	C18—C19	1.362 (4)
C1—C2	1.366 (3)	C18—C23	1.369 (4)
C1—Cl1	1.744 (2)	C18—Cl2	1.748 (3)
C2—C3	1.377 (3)	C19—C20	1.377 (3)
C2—H2	0.9300	C19—H19	0.9300
C3—C4	1.383 (3)	C20—C21	1.379 (3)
C3—H3	0.9300	C20—H20	0.9300
C4—C5	1.383 (3)	C21—C22	1.388 (3)
C4—C7	1.506 (3)	C21—C24	1.501 (3)
C5—C6	1.376 (3)	C22—C23	1.378 (3)
C5—H5	0.9300	C22—H22	0.9300
C6—H6	0.9300	C23—H23	0.9300
C7—C8	1.527 (3)	C24—C25	1.529 (3)
C7—H7A	0.9700	C24—H24A	0.9700
C7—H7B	0.9700	C24—H24B	0.9700
C8—O1	1.426 (2)	C25—O1	1.427 (2)
C8—C9	1.514 (3)	C25—C26	1.524 (3)
C8—H8	0.9800	C25—H25	0.9800
C9—C10	1.380 (3)	C26—C27	1.382 (3)
C9—C14	1.384 (3)	C26—C31	1.384 (3)
C10—C11	1.388 (3)	C27—C28	1.387 (3)
C10—H10	0.9300	C27—H27	0.9300
C11—O2	1.366 (2)	C28—O5	1.367 (2)
C11—C12	1.388 (3)	C28—C29	1.380 (3)
C12—O3	1.377 (2)	C29—O6	1.380 (2)
C12—C13	1.386 (3)	C29—C30	1.389 (3)
C13—O4	1.370 (2)	C30—O7	1.368 (3)
C13—C14	1.383 (3)	C30—C31	1.387 (3)
C14—H14	0.9300	C31—H31	0.9300
C15—O4	1.416 (3)	C32—O5	1.421 (3)
C15—H15A	0.9600	C32—H32A	0.9600
C15—H15B	0.9600	C32—H32B	0.9600
C15—H15C	0.9600	C32—H32C	0.9600
C16—O3	1.420 (3)	C33—O6	1.415 (3)
C16—H16A	0.9600	C33—H33A	0.9600
C16—H16B	0.9600	C33—H33B	0.9600
C16—H16C	0.9600	C33—H33C	0.9600
C17—O2	1.417 (3)	C34—O7	1.408 (3)
C17—H17A	0.9600	C34—H34A	0.9600
C17—H17B	0.9600	C34—H34B	0.9600
C17—H17C	0.9600	C34—H34C	0.9600
			
C6—C1—C2	121.2 (2)	C18—C19—H19	120.3
C6—C1—Cl1	118.53 (19)	C20—C19—H19	120.3
C2—C1—Cl1	120.29 (19)	C19—C20—C21	121.5 (2)
C1—C2—C3	119.0 (2)	C19—C20—H20	119.3
C1—C2—H2	120.5	C21—C20—H20	119.3
C3—C2—H2	120.5	C20—C21—C22	117.7 (2)
C2—C3—C4	121.5 (2)	C20—C21—C24	120.5 (2)
C2—C3—H3	119.2	C22—C21—C24	121.7 (2)
C4—C3—H3	119.2	C23—C22—C21	121.1 (2)
C3—C4—C5	117.7 (2)	C23—C22—H22	119.4
C3—C4—C7	121.6 (2)	C21—C22—H22	119.4
C5—C4—C7	120.7 (2)	C18—C23—C22	119.3 (2)
C6—C5—C4	121.2 (2)	C18—C23—H23	120.4
C6—C5—H5	119.4	C22—C23—H23	120.4
C4—C5—H5	119.4	C21—C24—C25	116.59 (18)
C1—C6—C5	119.4 (2)	C21—C24—H24A	108.1
C1—C6—H6	120.3	C25—C24—H24A	108.1
C5—C6—H6	120.3	C21—C24—H24B	108.1
C4—C7—C8	113.87 (18)	C25—C24—H24B	108.1
C4—C7—H7A	108.8	H24A—C24—H24B	107.3
C8—C7—H7A	108.8	O1—C25—C26	114.17 (16)
C4—C7—H7B	108.8	O1—C25—C24	106.46 (17)
C8—C7—H7B	108.8	C26—C25—C24	109.42 (17)
H7A—C7—H7B	107.7	O1—C25—H25	108.9
O1—C8—C9	113.29 (16)	C26—C25—H25	108.9
O1—C8—C7	106.12 (17)	C24—C25—H25	108.9
C9—C8—C7	109.84 (16)	C27—C26—C31	119.83 (19)
O1—C8—H8	109.2	C27—C26—C25	117.59 (18)
C9—C8—H8	109.2	C31—C26—C25	122.33 (19)
C7—C8—H8	109.2	C26—C27—C28	120.5 (2)
C10—C9—C14	120.14 (19)	C26—C27—H27	119.8
C10—C9—C8	120.19 (18)	C28—C27—H27	119.8
C14—C9—C8	119.42 (19)	O5—C28—C29	115.72 (19)
C9—C10—C11	119.61 (19)	O5—C28—C27	124.4 (2)
C9—C10—H10	120.2	C29—C28—C27	119.9 (2)
C11—C10—H10	120.2	O6—C29—C28	120.2 (2)
O2—C11—C12	115.27 (18)	O6—C29—C30	120.1 (2)
O2—C11—C10	124.19 (19)	C28—C29—C30	119.71 (19)
C12—C11—C10	120.54 (19)	O7—C30—C31	125.2 (2)
O3—C12—C13	120.50 (19)	O7—C30—C29	114.47 (19)
O3—C12—C11	119.92 (18)	C31—C30—C29	120.3 (2)
C13—C12—C11	119.33 (18)	C26—C31—C30	119.8 (2)
O4—C13—C14	124.71 (19)	C26—C31—H31	120.1
O4—C13—C12	115.09 (18)	C30—C31—H31	120.1
C14—C13—C12	120.18 (19)	O5—C32—H32A	109.5
C13—C14—C9	120.2 (2)	O5—C32—H32B	109.5
C13—C14—H14	119.9	H32A—C32—H32B	109.5
C9—C14—H14	119.9	O5—C32—H32C	109.5
O4—C15—H15A	109.5	H32A—C32—H32C	109.5
O4—C15—H15B	109.5	H32B—C32—H32C	109.5
H15A—C15—H15B	109.5	O6—C33—H33A	109.5
O4—C15—H15C	109.5	O6—C33—H33B	109.5
H15A—C15—H15C	109.5	H33A—C33—H33B	109.5
H15B—C15—H15C	109.5	O6—C33—H33C	109.5
O3—C16—H16A	109.5	H33A—C33—H33C	109.5
O3—C16—H16B	109.5	H33B—C33—H33C	109.5
H16A—C16—H16B	109.5	O7—C34—H34A	109.5
O3—C16—H16C	109.5	O7—C34—H34B	109.5
H16A—C16—H16C	109.5	H34A—C34—H34B	109.5
H16B—C16—H16C	109.5	O7—C34—H34C	109.5
O2—C17—H17A	109.5	H34A—C34—H34C	109.5
O2—C17—H17B	109.5	H34B—C34—H34C	109.5
H17A—C17—H17B	109.5	C8—O1—C25	115.55 (15)
O2—C17—H17C	109.5	C11—O2—C17	117.80 (17)
H17A—C17—H17C	109.5	C12—O3—C16	115.11 (17)
H17B—C17—H17C	109.5	C13—O4—C15	117.35 (17)
C19—C18—C23	121.0 (2)	C28—O5—C32	117.74 (18)
C19—C18—Cl2	119.7 (2)	C29—O6—C33	113.95 (19)
C23—C18—Cl2	119.3 (2)	C30—O7—C34	118.84 (19)
C18—C19—C20	119.4 (2)		
			
C6—C1—C2—C3	−2.4 (4)	Cl2—C18—C23—C22	−178.75 (19)
Cl1—C1—C2—C3	176.7 (2)	C21—C22—C23—C18	−0.8 (4)
C1—C2—C3—C4	0.8 (4)	C20—C21—C24—C25	94.9 (3)
C2—C3—C4—C5	1.2 (4)	C22—C21—C24—C25	−87.6 (3)
C2—C3—C4—C7	−177.0 (2)	C21—C24—C25—O1	72.5 (2)
C3—C4—C5—C6	−1.6 (3)	C21—C24—C25—C26	−163.64 (19)
C7—C4—C5—C6	176.6 (2)	O1—C25—C26—C27	−150.37 (18)
C2—C1—C6—C5	2.0 (4)	C24—C25—C26—C27	90.4 (2)
Cl1—C1—C6—C5	−177.09 (18)	O1—C25—C26—C31	35.4 (3)
C4—C5—C6—C1	0.1 (3)	C24—C25—C26—C31	−83.7 (2)
C3—C4—C7—C8	94.4 (3)	C31—C26—C27—C28	1.2 (3)
C5—C4—C7—C8	−83.7 (3)	C25—C26—C27—C28	−173.18 (19)
C4—C7—C8—O1	62.8 (2)	C26—C27—C28—O5	−178.8 (2)
C4—C7—C8—C9	−174.43 (18)	C26—C27—C28—C29	0.3 (3)
O1—C8—C9—C10	32.9 (3)	O5—C28—C29—O6	−4.0 (3)
C7—C8—C9—C10	−85.5 (2)	C27—C28—C29—O6	176.81 (19)
O1—C8—C9—C14	−152.82 (18)	O5—C28—C29—C30	177.58 (19)
C7—C8—C9—C14	88.7 (2)	C27—C28—C29—C30	−1.6 (3)
C14—C9—C10—C11	−0.2 (3)	O6—C29—C30—O7	3.1 (3)
C8—C9—C10—C11	174.02 (18)	C28—C29—C30—O7	−178.5 (2)
C9—C10—C11—O2	179.88 (18)	O6—C29—C30—C31	−176.9 (2)
C9—C10—C11—C12	−1.0 (3)	C28—C29—C30—C31	1.5 (3)
O2—C11—C12—O3	5.6 (3)	C27—C26—C31—C30	−1.3 (3)
C10—C11—C12—O3	−173.60 (18)	C25—C26—C31—C30	172.8 (2)
O2—C11—C12—C13	179.88 (18)	O7—C30—C31—C26	179.9 (2)
C10—C11—C12—C13	0.7 (3)	C29—C30—C31—C26	−0.1 (3)
O3—C12—C13—O4	−3.7 (3)	C9—C8—O1—C25	67.9 (2)
C11—C12—C13—O4	−177.92 (19)	C7—C8—O1—C25	−171.55 (16)
O3—C12—C13—C14	175.05 (19)	C26—C25—O1—C8	70.6 (2)
C11—C12—C13—C14	0.8 (3)	C24—C25—O1—C8	−168.52 (16)
O4—C13—C14—C9	176.62 (19)	C12—C11—O2—C17	−178.98 (19)
C12—C13—C14—C9	−2.0 (3)	C10—C11—O2—C17	0.2 (3)
C10—C9—C14—C13	1.7 (3)	C13—C12—O3—C16	89.9 (2)
C8—C9—C14—C13	−172.58 (19)	C11—C12—O3—C16	−95.9 (2)
C23—C18—C19—C20	0.2 (4)	C14—C13—O4—C15	2.8 (3)
Cl2—C18—C19—C20	179.3 (2)	C12—C13—O4—C15	−178.53 (19)
C18—C19—C20—C21	−0.3 (4)	C29—C28—O5—C32	−174.4 (2)
C19—C20—C21—C22	−0.1 (4)	C27—C28—O5—C32	4.8 (3)
C19—C20—C21—C24	177.6 (2)	C28—C29—O6—C33	88.4 (3)
C20—C21—C22—C23	0.6 (3)	C30—C29—O6—C33	−93.2 (3)
C24—C21—C22—C23	−177.0 (2)	C31—C30—O7—C34	−7.2 (4)
C19—C18—C23—C22	0.4 (4)	C29—C30—O7—C34	172.8 (2)
